# A new classification of upper gastrointestinal toxicity induced by immunotherapy: from endoscopic and pathological insights to clinical management

**DOI:** 10.1016/j.esmogo.2024.100083

**Published:** 2024-07-25

**Authors:** C. Casadio, L. Galvani, A. De Giglio, C. Casadei, M.L. Tardio, B. Melotti, F. Sperandi, F. Gelsomino, F. Comito

**Affiliations:** 1 Oncology Unit, IRCCS Azienda Ospedaliero-Universitaria di Bologna, Bologna; 2 Department of Medical and Surgical Sciences, University of Bologna, Bologna; 3 Gastroenterology Unit, Santa Maria delle Croci Hospital, Ravenna; 4 Pathology Unit, IRCCS Azienda Ospedaliero-Universitaria di Bologna, Bologna, Italy

**Keywords:** immune checkpoint inhibitors, upper gastro-intestinal toxicity, immune-related adverse events, oesophagogastroduodenoscopy

## Abstract

**Background:**

Immune checkpoint inhibitors (ICIs) have become the standard of care in several solid tumours. Thus the action of ICIs may lead to the development of inflammatory damage in nontumoral tissues, defined as immune-related adverse events (irAEs). Scanty data describe upper gastrointestinal tract toxicity.

**Patients and methods:**

We conducted a monocentric retrospective study, enrolling patients with advanced cancer, who developed histology-proven immune-related oesophago-gastro-duodenitis, treated with at least one cycle of ICI between January 2016 and November 2022.

**Results:**

We identified six patients with upper gastrointestinal irAEs: four affected by metastatic melanoma (three treated with nivolumab and one with nivolumab plus ipilimumab), one by unresectable cutaneous squamous cell carcinoma (treated with cemiplimab), and one by metastatic non-small-cell lung cancer (treated with pembrolizumab). Proton pump inhibitors and oral corticosteroids have been the mainstay of the management, and thus one patient had to receive intravenous methylprednisolone with hospitalisation, fasting, and parenteral nutrition. Based on the literature and our experience, we proposed a classification of ICI-induced upper gastrointestinal toxicity, with symptom and endoscopic sign grading. Each step of severity has been also correlated with a proposed diagnosis and clinical management.

**Conclusions:**

During ICI treatment, upper gastrointestinal symptoms can be a red flag for developing severe oesophago-gastro-duodenal toxicity that can impact patients’ quality of life and therapeutic plan. We recommend carefully investigating these symptoms, choosing a multidisciplinary approach to decide if an oesophagogastroduodenoscopy with random biopsy is indicated. [18]-fluoro-2-deoxy-d-glucose positron emission tomography/computed tomography might represent a promising complementary diagnostic tool. Steroids still represent the cornerstone of treatment, as for other irAEs.

## Highlights

• Upper GI toxicity caused by immunotherapy is a rare condition.
• In a retrospective study, 0.9% of 658 patients (NSCLC and melanoma) experienced upper GI issues.
• Symptoms encompassed nausea, vomiting, and weight loss. Treatment involved PPIs and steroids, showing rapid improvement.
• Endoscopic findings indicated erythematous mucosa, erosions, and atrophy.
• An evidence-based classification for upper gastrointestinal toxicity was proposed.

## Introduction

Cancer therapy has significantly changed following the advent of immune checkpoint inhibitors (ICIs). The use of programmed cell death protein 1 (PD-1) inhibitors, such as pembrolizumab or nivolumab, and cytotoxic T-cell associated protein-4 (CTLA-4) inhibitors, such as ipilimumab, has become the standard of care in several types of cancer, often leading to long-standing responses and considerable improvements in survival.^1^^,^^2^ The mechanism of action of these drugs is based on the blockade of inhibitory immune pathways, stimulating the immune system to recognise and attack cancer cells.^3^

Despite the significant benefits, the action of ICI may cause the occurrence of inflammation in normal tissues leading to the development of the so-called immune-related adverse events (irAEs). Notably, the toxicity profile of these agents can be dose limiting, lead to treatment discontinuation, and, in rare cases, even be fatal.

The most common irAEs occur in the skin (i.e. rash and itching), in the gastrointestinal (GI) tract (i.e. diarrhoea, hepatitis), and in ductless glands (i.e. hypothyroidism, hypopituitarism).^4^ Hence assessment and monitoring for signs and symptoms of the most common irAEs are mandatory, and regular blood tests are recommended.^5^

The median onset time for toxicity is ∼40 days, but it may vary considerably from an early occurrence during the first days after treatment administration to delayed onset up to 26 weeks.^6^ Usually, irAEs are managed with steroid therapy (0.5-1 mg per kg/daily), but refractory cases may require a higher load of steroids or biological treatment with other immunosuppressive agents.^7^

Although enterocolitis is the most studied form of GI toxicity, scanty data, mostly from case reports or small case series, are available on upper GI injury. As the treatment of ICI-related gastric toxicity differs from that of gastritis by other aetiologies, a proper differential diagnosis may be crucial in these patients.

This study describes epidemiological data, clinical manifestations, and management and outcomes of patients with irAEs affecting the oesophagus–GI tract and provides an evidence-based classification of ICI-induced upper GI toxicity with the associated multidisciplinary approach for diagnosis and clinical management.

## Patients and methods

### Study design and population

We conducted a single-centre retrospective study enrolling patients with non-small-cell lung cancer (NSCLC), melanoma, and nonmelanoma skin cancers and treated with at least one cycle of ICI between 1 January 2016 and 1 November 2022, who presented histology-proven immune-related oesophagus-gastro-duodenitis. We collected patients’ data from medical registries. The following variables have been collected: age, sex, comorbidities, concomitant therapies, tumour histology, anticancer treatments (type, dosage, prescription data), information and treatment of irAEs (type, dosage, prescription data), last follow-up, cause of death, and date of death. The clinical and ancillary data (laboratory tests, endoscopic tests, and instrumental tests) were extracted retrospectively by reviewing medical records.

## Results

The study included 658 patients who received at least one ICI infusion during the study period. Overall, 365 (55.4%) patients had NSCLC, 273 (41.4%) had melanoma, and 20 (3%) had nonmelanoma skin cancer.

Pembrolizumab was the most commonly administered ICI (39.0%), followed by nivolumab (37.2%), ipilimumab alone (9.1%), atezolizumab (8.6%), cemiplimab (3.0%), durvalumab (1.5%), and combination therapy with anti-PD-1 and anti-CTLA-4 inhibitors (0.9%).

We identified six (0.9%) patients with histology-proven immune-related upper GI toxicity induced by ICI. Among them, four patients were affected by metastatic melanoma (three cutaneous melanomas and one uveal melanoma) and treated with nivolumab (three patients) or the combination of nivolumab 1 mg/kg and ipilimumab 3 mg/kg (one patient); the patient with unresectable cutaneous squamous cell carcinoma received cemiplimab and the patient with metastatic NSCLC was treated with the combination of pembrolizumab and platinum-based chemotherapy.

The median latency period from the onset of ICI to symptom onset was 27 weeks (range, 12-43) and to endoscopic examination was 68 weeks (range, 12-124).

Table 1 presents the clinical and diagnostic findings of patients experiencing upper GI toxicity.

**Table 1:** Patients’ clinic and pathological characteristics

Characteristics	Patient #1	Patient #2	Patient #3	Patient #4	Patient #5	Patient #6
Age (years)	81	36	76	70	58	39
Sex	Male	Male	Female	Female	Male	Male
Pre-existing autoimmune disorder	Cutaneous toxicity	None	Cutaneous toxicity, pulmonary toxicity	Thyroid toxicity	Ocular toxicity	None
ECOG PS	1	0	0	0	0	0
Malignancy type	CSCC	Melanoma	Melanoma	Melanoma	Non-small-cell lung cancer	Uveal melanoma
Cancer stage	IV	IV	IV	IV	IV	IV
Symptoms	None	Nausea, vomiting, dyspepsia, bloating, heartburn, loss of appetite, and epigastric pain	Nausea, vomiting, dyspepsia, heartburn, and epigastric pain	Nausea, vomiting, heartburn, epigastric pain, and loss of appetite	Nausea, vomiting, heartburn, epigastric pain, and loss of appetite	Nausea, vomiting, heartburn, epigastric pain, loss of appetite, and weight loss
Time to symptoms onset	Asymptomatic	2.5 months	11 months	10 months	6 months	6 months
Immune checkpoint inhibitor	Cemiplimab	Nivolumab + ipilimumab	Nivolumab	Nivolumab	Pembrolizumab	Nivolumab
Gross OGD findings	Mild hyperaemic antral gastric mucosa	Diffusely hyperaemic gastric mucosa, with multiple erosions, detaching and bleeding spontaneously to the endoscope passage	Grade C oesophagitis according to the LA classification. Diffusely hyperaemic gastric mucosa. The duodenum with underrepresented folds and with visible fissures and a nodular appearance	Diffusely hyperaemic gastric mucosa	Hyperaemic and oedematous antral gastric mucosa. Oedematous duodenal bulb mucosa with a nodular appearance	Patchy hyperaemic gastric body mucosa. Intensely hyperaemic antral mucosa with the presence of copious mucus
Time to endoscopic evaluation^a^ (months)	30	3	11	11	8	31
Histopathology diagnosis	ICI-induced atrophic gastritis	ICI-induced severe gastroduodenitis	Oesophagitis and gastritis	ICI-induced ulcerative gastritis + autoimmune gastritis	ICI induced gastroduodenitis	Chronic atrophic gastritis
Histologic findings	⇑⇑ Plasma cells and CD3 T cells in the lamina propria, apoptotic figures in crypts	⇑⇑ Lymphocytes with the prevalence of CD8 T cells and eosinophils in the lamina propria	⇑⇑ Plasma cells, CD3 T cells, and eosinophils	⇑⇑ Lymphocytes with the prevalence of CD8 T cells, granulocytic fibrin material atrophy	⇑⇑ Lymphocytes with the prevalence of CD8 T cells	⇑⇑ Lymphocytes with the prevalence of CD8 T cells
^18^F-FDG PET/CT findings	Increased gastric uptake	Not carried out	No gastric uptake	Increased gastric uptake	Not carried out	Increased gastric uptake
Neutrophil-to-lymphocyte ratio at baseline	8.4	2.4	2.9	1.6	1.6	3.2
Disease response	CR	PR	CR	CR	PR	CR
Treatment	Already mild-dose steroids	High-dose steroids, oral and intravenous	Mild-dose steroids	High-dose steroids, oral	Mild-dose steroids	Metoclopramide and PPI

^18^F-FDG PET/CT, [18]-fluoro-2-deoxy-d-glucose (FDG) positron emission tomography (PET)/computed tomography; CR, complete response; CSCC, cutaneous squamous cells carcinoma; ECOG PS, Eastern Cooperative Oncology Group; OGD, oesophagogastroduodenoscopy; ICI, immune checkpoint inhibitor; LA Classification, Los Angeles Classification of oesophagitis; PPI, proton pump inhibitor; PR, partial response.

a From ICI first administration.

Approximately 2-4 weeks after the last ICI infusion, five out of six patients developed symptoms such as Grade 2-3 nausea according to the Common Terminology Criteria for Adverse Events^8^ (CTCAE version 5), vomiting (CTCAE Grade 2), unresponsiveness to standard antiemetic therapy (i.e. metoclopramide), along with epigastric pain, loss of appetite, and weight loss.

By contrast, one patient was clinically asymptomatic and [18]-fluoro-2-deoxy-d-glucose (FDG) positron emission tomography (PET)/computed tomography (CT) showed a gastric nonspecific uptake, subsequently investigated with an oesophagogastroduodenoscopy (OGD) that confirmed the diagnostic suspicion. Two patients continued treatment with ICI skipping 2 months of therapy.

For the symptomatic patients, other potential causes, such as immune-related endocrinopathies, were ruled out before carrying out OGD.

All patients were initially treated with a proton pump inhibitor (PPI) at a dose of 40-80 mg daily, which was continued even during steroid therapy. Four patients out of six were treated with systemic corticosteroids: oral prednisone 50 mg once a day (<1 mg per kg) was administered in three cases (Patients #3-#5; Table 1), tapering off after about 1 week; intravenous (i.v.) infusion of methylprednisolone 1 mg per kg/daily was administered to Patient #2 (Table 1). Indeed, this patient complained of Grade 3 nausea associated with Grade 3 vomiting, requiring hospitalisation and parenteral nutrition. At first, the patient did not respond to i.v. methylprednisolone at the dose of 0.5 mg/kg/daily, which was then increased to 1 mg/kg/daily, with tapering started after 7 days and weaned over in 4 weeks.

One patient (Patient #1; Table 1) was asymptomatic at diagnosis, even though he was under low-dose steroids (prednisone 5 mg per day) for analgesic purposes, whereas one patient had upper GI symptoms that were managed without the use of steroids, but only by using PPI (Patient #6; Table 1).

**Figure 1:**
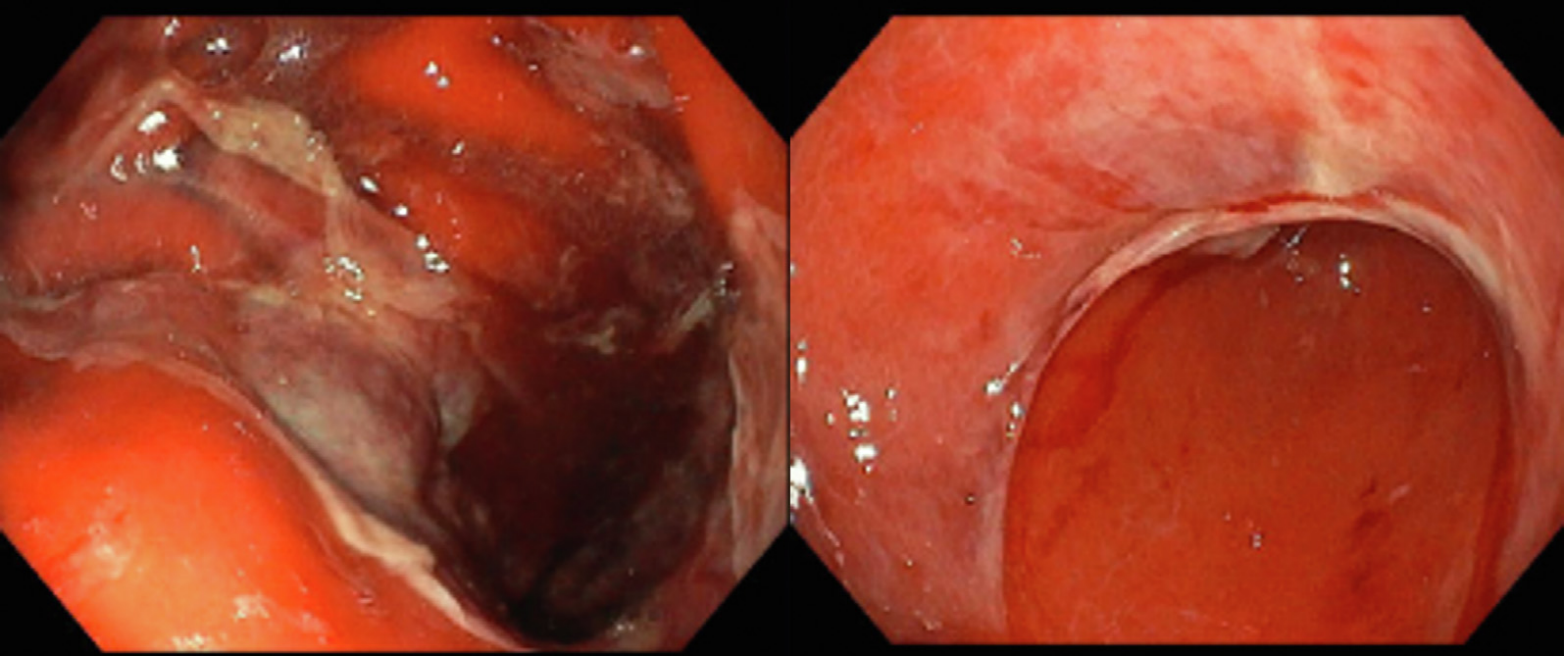
Severe acute lymphocytic gastritis with numerous apoptotic bodies and pathologic intraepithelial lymphocytosis, characterised by increased CD8+ T lymphocytes (red).

Overall, the symptoms rapidly improved within 24-48 h from the start of steroids. The patient who required hospitalisation and i.v. administration of steroids also underwent parenteral nutrition for 18 days due to severe clinical manifestation and endoscopic findings (Patient #2; Table 1, Figure 1). At the time of the endoscopic examination, three out of six patients did not take any chronic therapy (Patients #2, #4, and #6; Table 1), one patient took low-dose prednisone (Patient #1; Table 1), and none of the patients took PPIs and non-steroidal anti-inflammatory drugs (NSAIDs). None of the patients showed alterations in laboratory tests, particularly in blood cell count and formulas. Four out of six patients also showed other irAEs: three patients presented skin toxicities (vitiligo or rash; Patients #1-#3; Table 1), one patient thyroid toxicity (asymptomatic hypothyroidism; Patient #4; Table 1), and one patient developed Grade 1 pneumonitis according to the European Society for Medical Oncology (ESMO) Clinical Practice Guideline.^9^

### Endoscopic and histological evaluation

**Figure 2:**
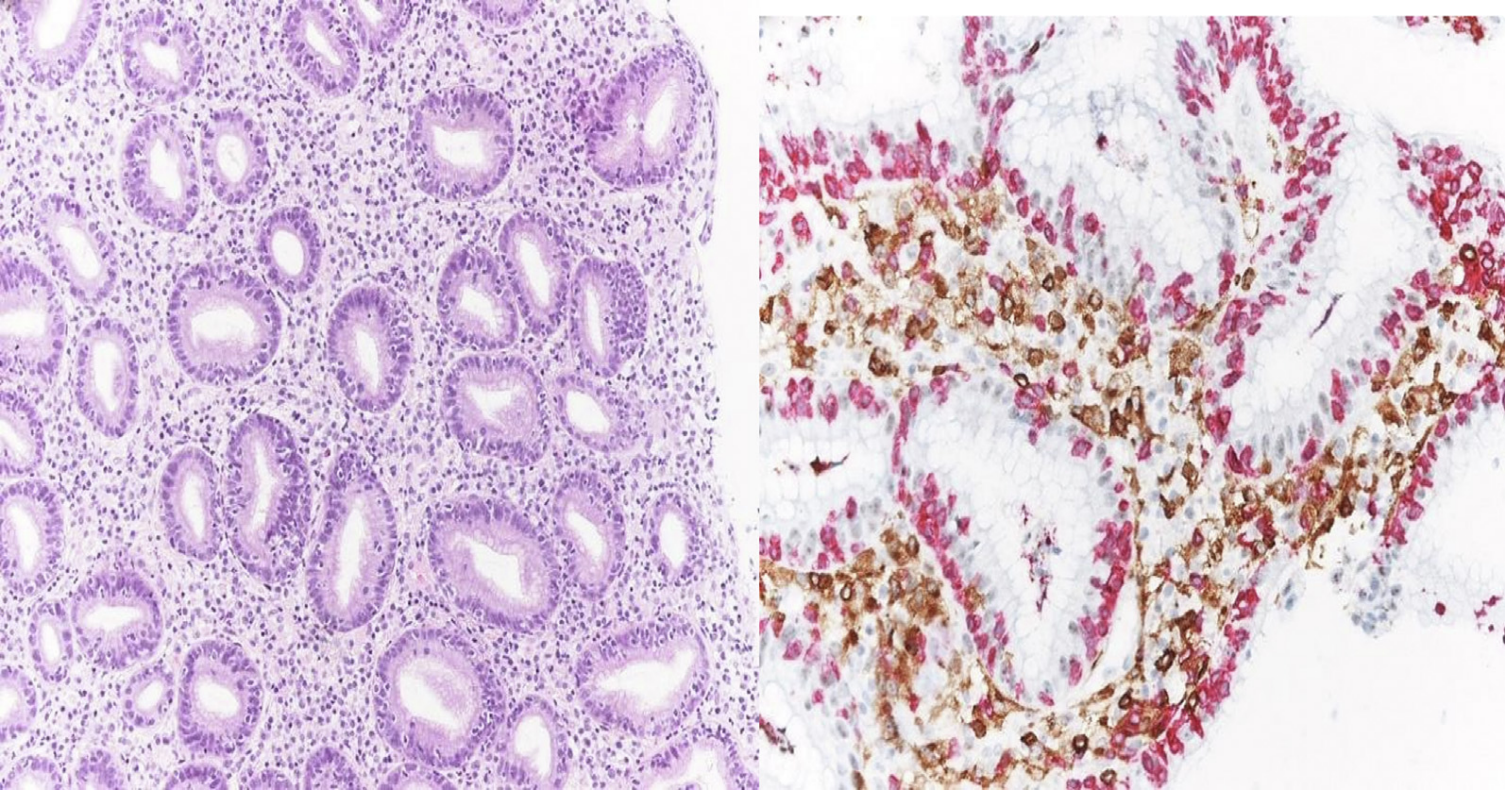
Severe and diffused stomach mucosa damage with erosions, fibrin, white exudate, mucous, and aspects of loss and detachment of the epithelium resulting in widespread bleeding.

All patients included in our analyses received an endoscopic evaluation. The endoscopic examination showed macroscopically diffuse erythematous mucosa of the stomach’s body as well as fundus and antrum with poorly represented folds (Supplementary Figure S1, available at https://doi.org/10.1016/j.esmogo.2024.100083). In two cases, the OGD also showed duodenal bulb and second duodenal portion involvement with inflammation, erosions, and hypo-atrophic duodenal mucosa. In one case, there were deep oesophageal confluent erosions involving <75% of the circumference in the middle and distal third of the oesophagus [Grade C oesophagitis according to Los Angeles (LA) classification]. In one patient, the whole stomach mucosa was diffusely de-epithelialised, with small patches of white inflammatory exudate and mucous adhesions, and spontaneous detachment of the epithelial flaps upon passage of the endoscopic instrument resulting in bleeding (Figure 2).

At histological examination, all cases showed severe and diffuse gastric disease. The gastric mucosa of the corpus and fundus showed severe atrophy of the oxyntic glandular component with pseudo pyloric metaplasia and lymphocytosis of the superficial and crypt epithelium with infiltration of neutrophils, eosinophils, plasma cells, and T lymphocytes cells (CD8+ prevalence). In two cases, there were also aspects of chronic ulcerative-erosive gastritis with atrophy of the glandular component and granulocytic fibrin material (Supplementary Figure S2, available at https://doi.org/10.1016/j.esmogo.2024.100083 and Figure 1). All cases showed no signs of infection from *Helicobacter pylori* (HP) or cytomegalovirus (CMV).

**Figure 3:**
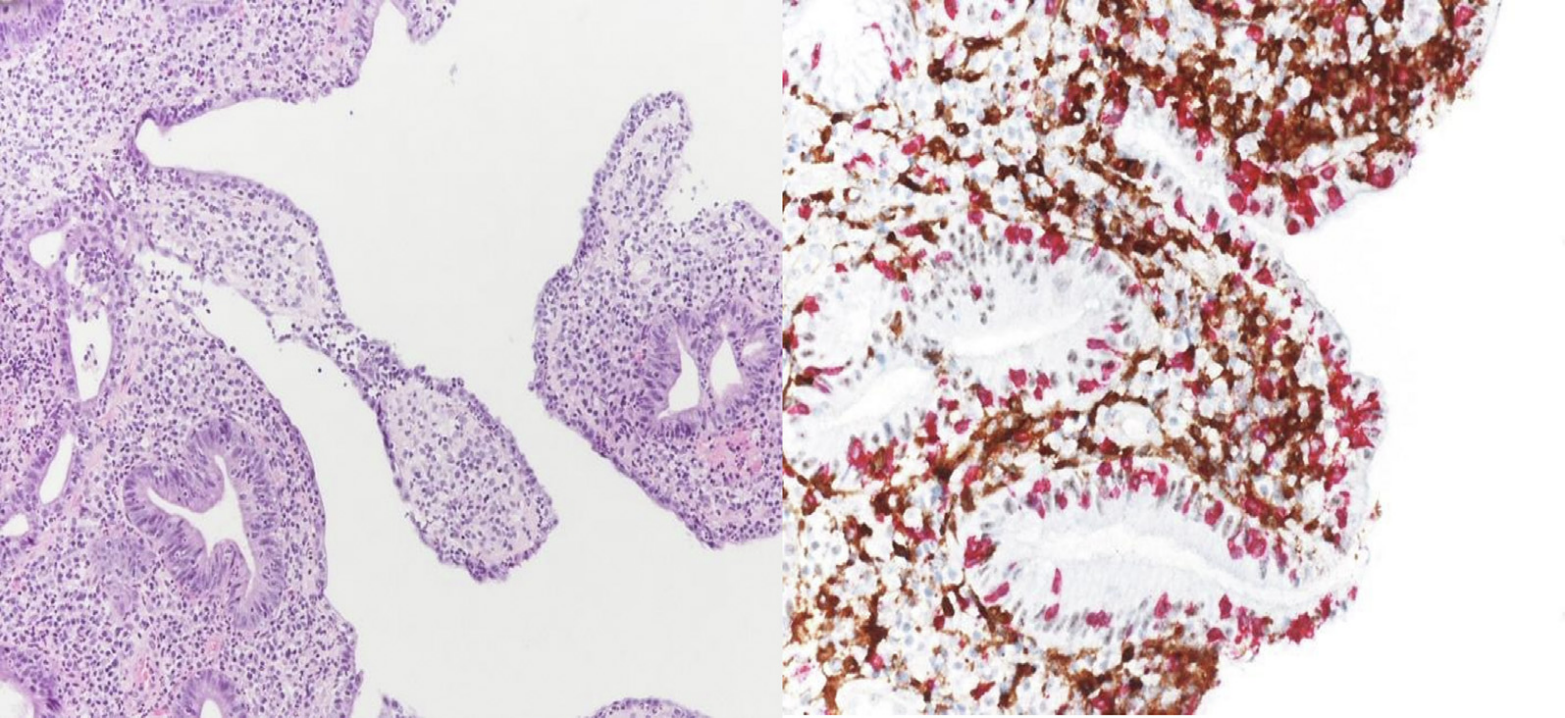
Partial villous atrophy with moderate inflammatory infiltrate in the lamina propria, comprising lymphoplasmacytic and granulocytic elements, along with pathologic intraepithelial lymphocytosis characterised by increased CD8+ T lymphocytes (red).

At the duodenal level, in two cases there was evidence of erosive duodenitis with severe lymphocytic infiltration with a prevalence of CD8+ lymphocytes in the epithelium surface, eosinophilic granulocytes in the lamina propria, apoptotic figures in the crypts, and glandular regenerative aspects (Supplementary Figure S3, available at https://doi.org/10.1016/j.esmogo.2024.100083 and Figure 3). In one case, the duodenum appeared normal under endoscopy examination, but the histological examination indicated duodenitis.

## Discussion

Therapy with ICI has led to an ever-increasing proportion of patients with a chronically controlled disease, thanks to a long-lasting response in multiple tumour types. A new spectrum of irAEs emphasises the need for multidisciplinary management and extensive data collection to treat them correctly. In this context, prompt recognition of adverse events is pivotal, requiring a careful physical examination, medical history, blood tests, imaging, and, in selected cases, invasive procedures.

In this study, we described clinical characteristics and diagnostic findings of upper GI ICI-related toxicity in a cohort of patients with cancer treated with immunotherapy over the past 6 years at our institution with an overall incidence rate of 0.9%.

The literature regarding GI irAEs focuses primarily on the toxicity of the lower GI tract, such as diarrhoea and colitis. Several case series described the correlation between ICI and upper GI toxicity, but none proposed evidence-based management guidance.^10^, ^11^, ^12^, ^13^

Tang et al.^14^ reported that 60 (1.2%) out of 4716 patients developed GI symptoms. Interestingly, 38 out of 60 patients underwent both OGD and colonoscopy, and 21 patients (34%) had concurrent upper and lower GI tract involvement. Moreover, they analysed other risk factors for gastritis, such as chemotherapy, radiotherapy, and recent use of NSAIDs, observing a similar rate of ulceration between the cohorts with and without other risk factors and an independent association between ICI use and upper GI inflammation. In our cohort, none of the patients received a colonoscopy because of the absence of symptoms or signs of concurrent lower GI involvement. Considering other risk factors of gastric inflammation, none of our patients underwent radiotherapy or NSAID treatment and only one patient received concurrent chemotherapy.

The median latency period from the onset of ICI to symptom onset was 27 weeks (range 12-43 weeks). The time between the ICI start and the endoscopic diagnosis was heterogeneous, ranging from 3 to 31 months. Although the symptoms were more timely, all patients continued the immunotherapy and underwent endoscopic evaluation even after the symptoms persisted. Two out of six patients had already completed immunotherapy treatment: one had stopped immunotherapy the month before the symptoms appeared, whereas the other one was asymptomatic but had gastric uptake on ^18^F-FDG PET/CT 3 months after stopping immunotherapy. The symptoms observed among our patients were similar to those in the previously mentioned studies, mainly comprising nausea, vomiting, dyspepsia, heartburn, and bloating.

Interestingly, four out of six patients achieved a complete response and the others achieved a partial response to ICI. Endoscopic findings were comparable with those previously described, varying from mucosal erythema to severe ulceration and bleeding in the most severe cases. In clinical practice, oesophageal biopsies are not routinely carried out in cases of uncomplicated erosive oesophagitis (endoscopic finding compatible with oesophageal reflux oesophagitis), given their limited usefulness for diagnostic and therapeutic purposes. Instead, in selected patients, being treated with ICI and having characteristic symptoms, we also recommend carrying out biopsies of the oesophagus as it could be affected, either alone or in conjunction with the stomach and duodenum. Indeed, Panneerselvam et al.^15^ observed that out of 657 patients who underwent OGD during ICI-based therapy, 21 (3%) had oesophagitis, mostly from anti-PD-1 and anti-PD-L1. The majority of patients had middle–distal-third oesophageal distribution and concurrent gastric involvement, while only three patients had isolated oesophageal disease. At the histological examination, we observed increased lymphocytes, plasma cells, and neutrophil and eosinophil counts in the lamina propria, with inflammatory distortion of the crypts, oedema, and vascular congestion as reported in other studies.^14^, ^15^, ^16^ In particular, histological lymphocytosis with prevalent CD8+ infiltrate might be a characteristic finding, as Fujii et al.^17^ reported. It is also important to histologically exclude a CMV or HP infection, as these do not respond to steroid therapy.

In our cohort, the primary treatment approach consisted of PPI administration (100%), supplemented with steroids (50%), tailored to the severity of symptoms and endoscopic findings following thorough multidisciplinary discussion. Only one patient was treated exclusively with PPI and metoclopramide with sustained benefit. Interestingly, even in the studies by Tang et al.^14^ and Panneerselvam et al.,^15^ PPIs were the primary treatment administered, followed by steroids and H2 blockers. Furthermore, in 2022 Homicsko et al.^18^ investigated the association between PPI and ICI using data from the CheckMate 066, 067, and 069 studies, finding insufficient evidence to conclude that PPIs affect the efficacy of ICIs.

As a result of GI toxicity, two patients discontinued immunotherapy permanently, while two others continued treatment with ICIs alongside chronic PPI therapy. However, they reported persistent epigastric pain and heartburn, symptoms consistent with gastroesophageal reflux disease.

ESMO guidelines for the management of immunotherapy toxicity briefly deal with this topic, recommending PPI therapy in most cases while reserving steroidal or biologic immunosuppressive therapy for severe forms with deep gastric ulcerations, without giving indications on the management of ICI therapy.^15^ The decision to reintroduce ICIs after GI toxicity should be made on a case-by-case evaluation. In the context of planned ICI resumption, concurrent use of low-dose steroids or nonsteroidal immunosuppressants may minimise the risk of further recurrence of toxicity. However, these recommendations are based on the evidence of lower GI toxicity, but a standardised approach for the clinical management of upper GI toxicity is not yet defined.

Remarkably, a longitudinally shared classification of upper GI irAEs is not currently available. CTCAE 5.0,^8^ a set of criteria formulated to grade and manage adverse effects of cancer therapy, provides a general classification of gastritis, ranging from Grade 1, with asymptomatic patients not requesting intervention, to more severe symptoms such as dysphagia and anorexia demanding clinician intervention or hospitalisation, to Grade 5 (drug-related death). However, this classification may be partially exhaustive, as it does not consider the endoscopic findings and the treatment algorithm. The absence of a specific classification and extensive recommendations for upper GI irAEs from international oncology societies may hamper the proper diagnostic work-up and management, especially outside of tertiary referral hospitals.

Systemic steroid therapy is the established mainstay for irAEs,^6^^,^^9^ and a growing body of evidence is leading to the knowledge that intercurrent steroid administration does not significantly undermine ICI efficacy.^9^^,^^19^^,^^20^ Moreover, the association between irAEs and survival outcomes of patients receiving immunotherapy for advanced solid malignancies has been extensively described regardless of histotype.^21^^,^^22^ Interestingly, in our cohort, all patients with upper GI toxicity showed a complete or partial response to ICI.

**Figure 4:**
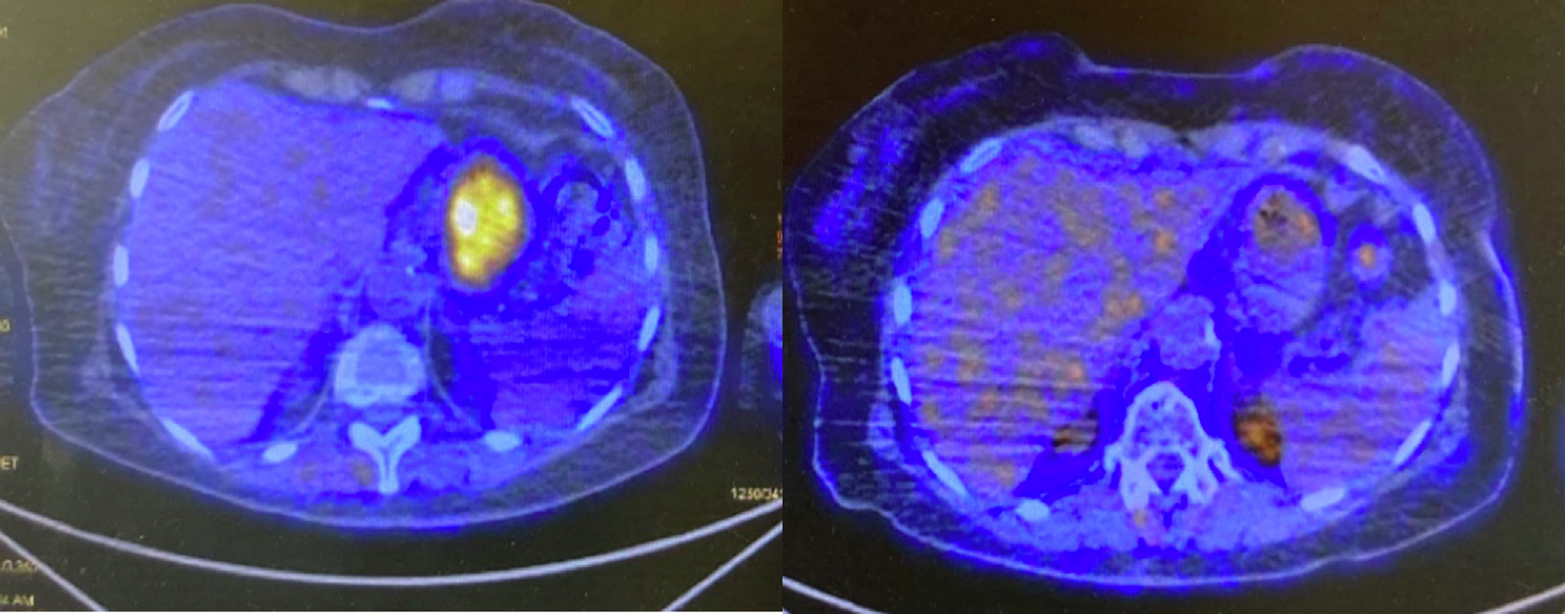
Patient #4 before and after steroid therapy.

Cherk et al.^23^ observed that ^18^F-FDG PET/CT scanning, now increasingly used for staging and restaging of disease, might be a promising diagnostic tool in irAEs management. Indeed, ^18^F-FDG PET/CT scanning could show mild to moderately increased diffuse ^18^F-FDG uptake, which could correlate with irAEs, thus identifying them early or could be used to confirm a clinically suspected irAE. Intriguingly, 2 out of 5 patients showed diffuse gastric uptake, even in asymptomatic or paucisymptomatic cases. Thus, further investigations, such as OGD, should be discussed in patients with suspected gastric uptake treated with ICI (Supplementary Figure S4, available at https://doi.org/10.1016/j.esmogo.2024.100083 and Figure 4).

However, it might be reasonable, in patients undergoing immunotherapy with evidence of diffuse gastric uptake on ^18^F-FDG PET/CT even if asymptomatic, to carry out an OGD to rule out other causes and to set up, in case of a histological confirmation, an adequate therapy before a possible worsening of the clinical conditions. Finally, an endoscopic follow-up might be useful to monitor the response patterns.

### Management of ICI-induced upper GI toxicity

Based on a review of the existing literature and our experience at our academic hospital, we have developed an evidence-based classification for ICI-induced upper GI toxicity, along with a connected multidisciplinary approach for diagnosis and clinical management. In cases of clinically suspected or endoscopically established gastric toxicity, we consistently recommend avoiding NSAIDs, if necessary, eradicating HP infection.

The mild toxicity (Grade 1) characterises asymptomatic patients with incidental findings on abdominal imaging (PET-FDG) or paucisymptomatic patients with mild epigastric pain, nausea, heartburn, and bloating.

The endoscopic scenario might vary from normal oesophageal mucosa to patchy hyperaemic mucosa with Grade A or B erosive oesophagitis (according to the LA classification) and patchy or diffuse gastric mucosal erythema and oedema with usually a regular duodenal mucosa. The recommended treatment for mild toxicity is PPI and metoclopramide as needed without impacting the immunotherapy course. OGD and oral steroid therapy may be appropriate if not responsive to antiemetics and PPI conservative treatment.

Moderate toxicity (Grade 2) is associated with nausea, moderate to severe epigastric pain and heartburn, mild dysphagia, and anorexia with scarce weight loss (between 5% and 10% of the patient’s total weight in the past 6 months not taking into account the patient’s total body mass index). On an endoscopic level, oesophageal mucosa is diffusely hyperaemic and oedematous, and fragile at endoscope contact, with initial white inflammatory exudate and with erosive oesophagitis Grade B or C (according to the LA classification). Gastric mucosa can be extensively affected by erosions, also fragile at endoscope contact with duodenal mucosa presenting erythema, oedema, and surface erosions. The recommended treatment for moderate toxicity (Grade 2) is PPI, oral prednisone (0.5-0.8 mg/kg/day), metoclopramide as needed, and withholding of ICI therapy. A low steroid dose and ICI rechallenge should be considered after the evidence of clinical and endoscopic resolution. At last, severe upper GI toxicity (Grades 3-4) might present with repeated vomiting, weight loss, severe epigastric pain, and severe dysphagia to solid food or liquids. In addition, melena, hematemesis, and acute anaemia might occur. The endoscopic findings can be represented by diffuse erosive oesophagitis with multilocation involvement, usually Grade D (according to the LA classification) as well as multiple ulcers. Intensely hyperaemic mucosa, with erosions and aspects of loss and detachment of mucosa with widespread spontaneous bleeding, is usually observed at the gastric level. Besides, the presence of multiple ulcers, small patches of white inflammatory exudate, and mucous adhesions might be observed. The duodenum can be characterised by intense loss of villi with erosions or ulcers and consequent loss of regular duodenal folds with visible fissures and nodular appearance. The severe toxicity (Grades 3-4) may demand hospitalisation to intensify the supportive care. The recommended treatment for severe toxicity (Grades 3-4) is high-dose PPI (bis in die), i.v. methylprednisolone (1-2 mg/kg), fluid support, and metoclopramide as needed. Moreover, fasting and parenteral nutrition are recommended. In this context, ICI discontinuation should be considered (Table 2). The use of biological drugs (i.e. infliximab) should be considered in patients who do not respond to high-dose i.v. steroids or in patients with concomitant severe immune-related colitis.

**Table 2:** Evidence-based classification and management for ICI-induced upper GI toxicity^a^

Grade	Clinical presentation	Time to OGD	Endoscopic findings	Treatment
1—Mild	Asymptomatic: casual findings on abdominal imaging. Paucisymptomatic: mild epigastric pain, nausea, heartburn, early feeling of fullness or satiety.	OGD if not responsive to conservative treatment with antiemetics and proton pump inhibitors.	Oesophagus: usually normal mucosa, but patchy hyperaemic mucosa might also be present; Grade A^a^ or B erosive oesophagitis. Stomach: diffusely hyperaemic, oedematous mucosa. Duodenum: usually has a regular-looking mucosa with normally represented villi	Continue ICI PPI 40 mg/day Antiemetic Eradication of HP infection Limit the use of NSAIDs Consider oral prednisone 0.5-08 mg/kg if no response to PPI and antiemetic therapy but if the diagnosis is confirmed by OGD
2—Moderate	Nausea, moderate to severe epigastric pain/heartburn, and anorexia. Initial weight loss might occur.	OGD recommended Second-look OGD might be recommended	Oesophagus: diffuse hyperaemic and oedematous mucosa and erosive oesophagitis (Grade B or C). Stomach: the mucosa is extensively affected by erosions and is fragile to the passage of the endoscope. Duodenum: The mucosa appears hyperaemic with some erosions, with initial signs of rarefaction of the villi and squat villi.	Withhold ICI^b^ PPI 40 mg/day Prednisone oral 0.5-0.8 mg/kg Antiemetic Eradication of HP infection Limit the use of NSAIDs Exclude CMV infection
3/4—Severe	Vomiting, weight loss, and severe epigastric pain. Inability to eat and, in some cases, to drink liquids. Melena, hematemesis, and acute anaemia might occur. Hospitalisation is usually required.	OGD recommended Second-look OGD recommended	Oesophagus: diffuse erosive oesophagitis with multilocation involvement, usually Grade D. Also ulcer might be present. Stomach: the mucosa appears intensely hyperaemic, with erosions, and aspects of loss and detachment with widespread spontaneous bleeding. The presence of multiple ulcers with small patches of white inflammatory exudate and abundant mucous adhesions might also be observed. Duodenum: characterised by intense loss of the normal appearance of the villi with erosions and possible ulcers and consequent loss of regular duodenal folds.	ICI discontinuation Fasting PPI 40 mg twice a day Methylprednisolone (intravenous) 1-2 mg/kg Consider biological drugs (e.g. infliximab) Liquids i.v. Antiemetic Consider parenteral nutrition Eradication of HP infection Exclude CMV infection Limit the use of NSAIDs

CMV, cytomegalovirus; OGD, oesophagogastroduodenoscopy; HP, *Helicobacter pylori*; ICI, Immune checkpoint inhibitor; i.v., intravenously; NSAID, non-steroidal anti-inflammatory drug; PPI, proton pump inhibitor.

a According to the Los Angeles (LA) classification oesophagitis: Grade A, one (or more) mucosal break no longer than 5 mm that does not extend between the tops of two mucosal folds; Grade B, one (or more) mucosal break more than 5 mm long that does not extend between the tops of two mucosal folds; Grade C, one (or more) mucosal break that is continuous between the tops of two or more mucosal folds but which involves <75% of the circumference; Grade D, one (or more) mucosal break which involves at least 75% of the oesophageal circumference.

b Consider ICI rechallenge with low-dose steroids after the evidence of clinical and endoscopic resolution.

Furthermore, a second endoscopic evaluation is strongly recommended for patients with severe endoscopic findings (Grades 3-4) to assess the response to systemic steroid therapy. This is particularly important in cases where symptoms recur upon tapering or discontinuation of steroid therapy and for patients receiving biological drugs. It is advisable to achieve at least endoscopic remission, and preferably histological remission, before considering suspension of biological drugs due to the high risk of recurrence. We also recommend a second endoscopic evaluation for all patients who have discontinued ICI therapy due to toxicity but require resumption. Achieving endoscopic remission before rechallenge is advisable.

We do not recommend a second endoscopic evaluation in patients with endoscopic findings of mild–moderate toxicity (Grades 1 and 2) with excellent response to PPIs or oral steroids and complete resolution of symptoms or in patients with poor life expectancies.

As we have shown in Table 1, five out of six patients presented a low neutrophil-to-lymphocyte ratio at baseline, and we did not evaluate the related trends, but, as pointed out in some previous studies,^24^ these data could be useful as a dynamic marker to evaluate the severity of irAEs and subsequent prognosis.

Finally, as per irAEs toxicity guidelines, multidisciplinary management is recommended, with a toxicity board playing a crucial role in referral centres where patients should ideally receive treatment.

Our study has several limitations worthy of discussion. First, an underestimation of mild forms of IR gastritis should be considered, as many mild symptomatic patients did not undergo endoscopic evaluation and were potentially excluded, which contributed to the lower incidence registered in our analysis. Furthermore, as a result of the retrospective nature of the study, the diagnostic work-up and management were heterogeneous and physician based. Moreover, the following points should be further investigated within larger and prospective studies: a clinical–pathological classification of severity, indication and timing of endoscopy at diagnosis and during follow-up, the role of ^18^F-FDG PET-CT, and an established medical treatment algorithm including the management of immunotherapy. Nevertheless, it is crucial to emphasise that the decision to rechallenge with ICIs must be evaluated individually by an experienced multidisciplinary team. Factors to consider are therapeutic alternatives, the patient’s previous oncological response to ICI, and the severity of upper GI involvement.

### Conclusion

During ICI treatment, upper GI symptoms can be a red flag for developing severe upper GI toxicity that can impact patients’ quality of life and treatment decision making. We recommend conducting a thorough investigation of these symptoms and convening a multidisciplinary discussion for each case to determine whether an OGD with random biopsies from all areas of the upper GI tract (oesophagus, stomach, duodenum) is warranted. ^18^F-FDG PET/CT might represent a promising complementary diagnostic tool. Steroid therapy still represents the cornerstone of treatment, as for other immune-related toxicities.
