# Supplementary material for: A new classification of upper gastrointestinal toxicity induced by immunotherapy: from endoscopic and pathological insights to clinical management
Source: ESMO Gastrointest Oncol. 2024 Jul 25;5:100083. doi: 10.1016/j.esmogo.2024.100083 (PMC12836540; doi:10.1016/j.esmogo.2024.100083)
Supplement: Supplementary Figures S1-S4 [file mmc1.docx]

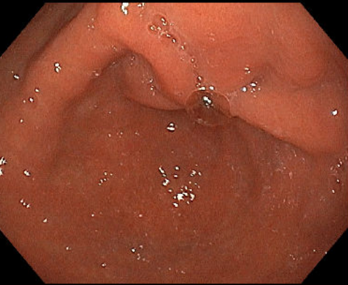


***Figure 1S.*** Antrum mucosa showing mild inflammation signs such as hyperaemic and edematous mucosa.


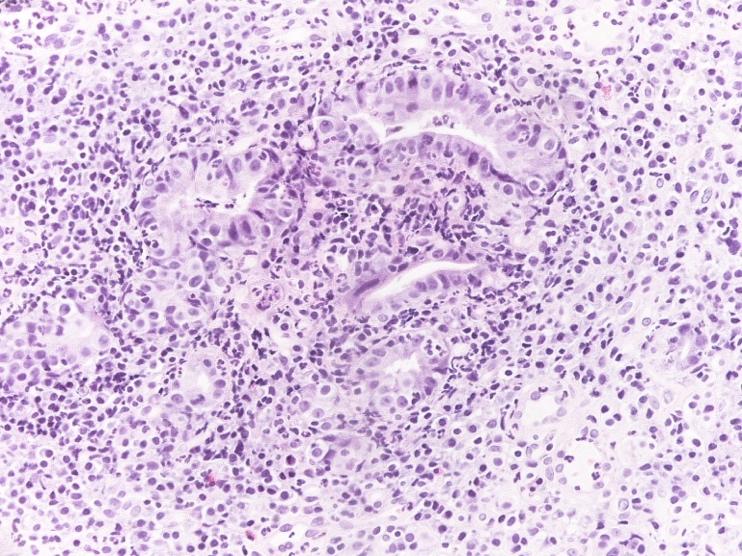


***Figure 2S.*** Severe acute and erosive gastritis with numerous apoptotic bodies.


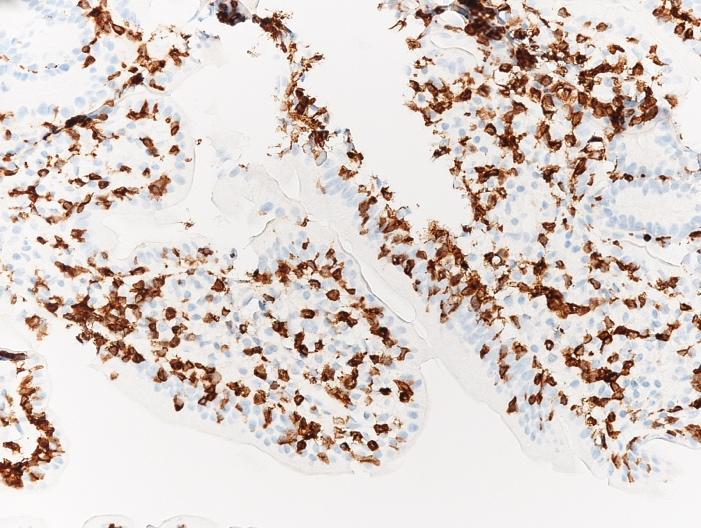

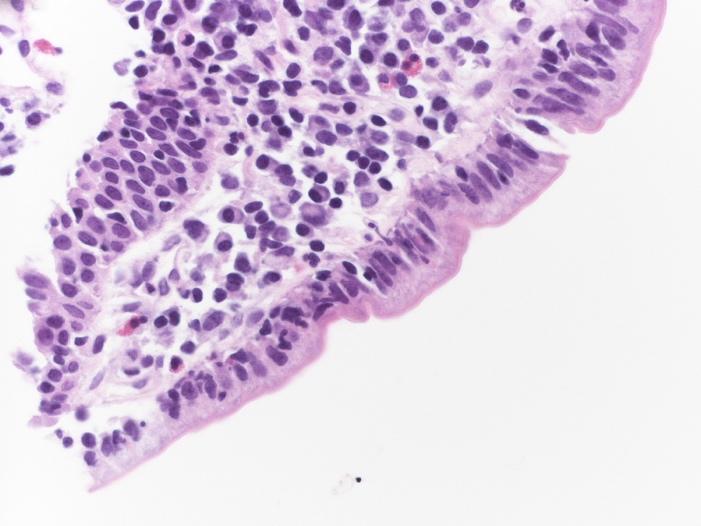


***Figure 3S.*** Histological picture showing a duodenal villus with increased number of intraepithelial lymphocytes (more than 25x100 enterocytes), well evidenced with anti-CD3 immunostaining, and apoptotic bodies.


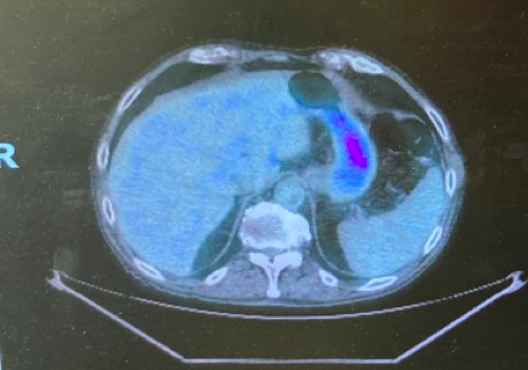

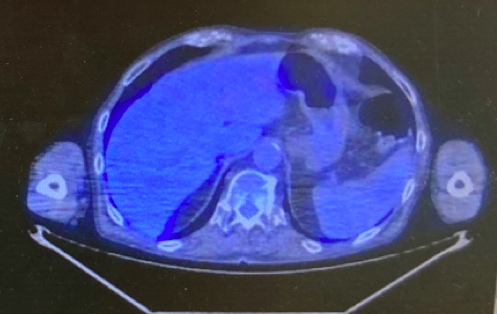
***Figure 4S.*** Patient #1 before and after steroid therapy.
